# Vertical Jump Neuromuscular Performance Characteristics Determining On-Court Contribution in Male and Female NCAA Division 1 Basketball Players

**DOI:** 10.3390/sports11120239

**Published:** 2023-12-04

**Authors:** Nicolas M. Philipp, Dimitrije Cabarkapa, Ramsey M. Nijem, Stryder D. Blackburn, Andrew C. Fry

**Affiliations:** Jayhawk Athletic Performance Laboratory—Wu Tsai Human Performance Alliance, Department of Health, Sport and Exercise Science, University of Kansas, Lawrence, KS 66045, USA; dcabarkapa@ku.edu (D.C.); coachnijem@ku.edu (R.M.N.); stryder.blackburn@ku.edu (S.D.B.); acfry@ku.edu (A.C.F.)

**Keywords:** force, power, sport, playing time, athlete, testing, monitoring

## Abstract

While various quantifiable physical attributes have been found to contribute to athletes’ performance, there is a lack of scientific literature focused on examining how they relate to success during competition performance. The aim of this study was to investigate different countermovement jump (CMJ)-derived force–time characteristics and their utility in distinguishing high from low performers within a measure of on-court contribution (i.e., minutes per game played). Twenty-nine collegiate athletes (n = 15 males and n = 14 females) volunteered to participate in this investigation and performed CMJs on dual force plates sampling at 1000 Hz, weekly over the course of their basketball season. The athletes’ average of their three best test-days across the season was used for further analysis. To identify their on-court contribution, athletes were divided into groups with high and low minutes per game, based on a median-split analysis. The findings suggest that at the overall group level (i.e., both genders), the modified reactive strength index (mRSI) and braking rate of force development (RFD) revealed the greatest between-group magnitudes of difference, with athletes playing more minutes per game showing greater performance. At the team-specific level, the braking RFD, average braking velocity, and mRSI were shown to be the greatest differentiators between groups for the men’s team. The women’s high-minutes group displayed greater magnitudes of mRSI and jump height. By identifying the neuromuscular qualities seen in top performers within their respective populations, the attributed physical performance underpinning these qualities may be identified, providing practitioners with insights into physical performance qualities and training methodologies that have the potential to influence basketball performance.

## 1. Introduction

Globally, basketball is one of the most popular sports. Determinants of success within this court-based sport are multifaceted and involve different quantifiable measures at the team and individual levels. From a physical perspective, the sport requires athletes to show proficiency in a number of motor abilities such as speed, strength, and endurance [1]. Such qualities have been quantified through a number of different assessments, such as shuttle or repeat sprint tests; vertical jump tasks; and dynamic and isometric, upper- and lower-body strength tests [2,3]. At the more sport-specific team level, it has been proposed that variables that are able to determine overall victory include defensive rebounds, assists, steals, and committed fouls, as well as two-point, three-point, and free-throw shooting accuracy [4]. In a recently published study, Cabarkapa et al. (2022) suggested that at the National Basketball Association (NBA) level of play, the field goal percentage and defensive rebounding are the top two game-related statistical parameters capable of discriminating winning from losing game outcomes [5]. Therefore, in order for teams to secure the desired game outcome, individual players need to show proficiency within most of the previously mentioned variables. While in most cases, performance in such variables is primarily underpinned by proficiency in related sport-specific skills, previous research has documented a positive relationship between various physical performance attributes and sport-specific outcomes [6,7,8]. For example, Hoffman et al. (1996) previously highlighted the relationships of maximal lower-body strength, vertical jump performance, speed, and agility with playing time among male National Collegiate Athletic Association (NCAA) Division-I basketball players [6]. On the other hand, Lockie et al. (2020) recently found no significant relationships between playing time and NBA combined physical performance tests (e.g., countermovement vertical jump, lane agility drill, 83.91 kg bench press) within Division-I mid-major basketball players [7]. In addition, Dawes et al. (2016) suggested that pre-season strength levels within the bench press and back squat exercise were related to in-season playing times within a group of NCAA Division-II basketball players [8]. In a similar investigation, Cabarkapa et al. (2020) found that lower-body strength and power were positively associated with post-collegiate playing opportunities, when examining a cohort of collegiate athletes over a seven-year time span [9]. Lastly, from a physical performance standpoint, Torres-Ronda et al. (2016) found that vertical jump capacity was positively correlated with playing time within a sample of 13–14-year-old Spanish basketball players [1].

While the aforementioned research reports highlight the positive association between some of the key physical attributes (e.g., lower-body strength) and on-court basketball performance, there is still a lack of scientific literature focused on identifying physical performance indicators as they related to sport-specific measures (e.g., playing time). Through the growing availability of portable and affordable sport science technology, more sports scientists and strength and conditioning practitioners have gained access to devices such as force plates to track and record different features of athletic performance. Specifically, the use of force plates to measure ground reaction forces and force–time curve characteristics during dynamic and isometric tasks has increased in use and popularity amongst sports science practitioners [10]. Within basketball settings, force plates are primarily being used as a tool in screening, monitoring, profiling, and rehabilitating athletes [11]. According to Schuster et al. (2020), the majority of force plate testing in elite sports is centered around the countermovement jump (CMJ) [11]. The CMJ is a common way to non-invasively measure an athlete’s use of the stretch-shortening cycle (SSC). The SSC describes a phenomenon consisting of an eccentric phase or stretch followed by an isometric transitional period (i.e., amortization phase), leading into an explosive concentric action [12]. From the CMJ, practitioners commonly extract and analyze force–time metrics such as the jump height (JH), modified reactive strength index (mRSI), impulse (IMP), peak power (PP), and average power (AP), as well as the rate of force development (RFD) and rate of power development (RPD) across the concentric and eccentric phases of the jump [13]. Such metrics may be used to monitor athletes’ neuromuscular fatigue and readiness over time and to track progress and individualize the approach toward the implementation of training strategies [14]. However, in order to understand which metrics might be of interest with regards to the individualization of training, coaches and other practitioners require a thorough understanding of which force–time metrics are related to success within the sport. Similar approaches to the ones highlighted previously are needed to gain a better understanding of the relationship between different force–time metrics and on-court performance within basketball.

With this idea in mind, the primary aim of this study was to investigate different force–time characteristics, extracted from the CMJ test, and measures of on-court contribution, as quantified by the minutes per game played by male and female collegiate basketball players. By identifying the neuromuscular qualities seen in top performers within their respective populations, the physical attributes underpinning these qualities may be identified, providing practitioners with closer insights into physical performance qualities and training methodologies that have the potential to influence on-court basketball performance.

## 2. Materials and Methods

### 2.1. Experimental Approach to the Problem

In line with the research question proposed in the present study, a cross-sectional research design was used, utilizing CMJ testing data from the respective teams throughout the in-season competitive period (i.e., November–March). Data were collected on a near-weekly basis as part of the team’s regular strength and conditioning sessions in the weights room. Prior to the collection of jump data, all athletes performed a brief dynamic warm-up consisting of a number of dynamic stretching exercises. The CMJ testing was performed at the beginning of each training session to minimize the possible influence of fatigue. The athlete’s average of their three best test-days across the season was used for further analysis. This was done to generate a value reflecting true maximal performance. The authors observed no specific tendency in the times of the season when the three best test-days occurred, often reflecting time-points from different parts of the in-season period. To identify the on-court contribution, within each team, athletes were divided into groups of high and low minutes per game, based on a median-split analysis. The strength and conditioning coaches of each team confirmed that the group split generally reflected a distinction between those athletes in the primary playing rotation of the team and those athletes who are either non-scholarship players or players who are rarely part of the regular playing rotation.

### 2.2. Participants

The sample for this study consisted of 29 NCAA Division-I basketball players from a Power 5 university: 15 men’s basketball players (age = 20.9 ± 1.8 years, height = 199 ± 9.5 cm, body mass = 96.9 ± 11.6 kg) and 14 women’s basketball players (age = 20.9 ± 1.23 years, height = 185 ± 8.9 cm, body mass = 86.6 ± 16.4 kg). All testing procedures were approved by the University’s Institutional Review Board, and all participants provided their written informed consent.

### 2.3. Countermovement Jump Test

Following the warm-up procedures, athletes performed a total of three CMJs. Each jump was separated by a 15–30 s rest interval. The data were collected using Hawkin Dynamics unidimensional dual force platforms (Hawkin Dynamics, Westbrook, ME, USA) sampling at 1000 Hz. Force plates were zeroed/calibrated prior to data collection. Athletes were instructed to step onto the force plate, stand still with their hands on their hips for a total of two to three seconds, and then jump as fast and as high as possible while keeping their hands on their hips throughout the entire movement. More specifically, a visual and auditory signal from a tripod-mounted tablet in front of the athlete was used to initiate data collection. Strong verbal encouragement was provided for each jump through all testing procedures to ensure that maximal effort was given during each jump.

**Figure 1:**
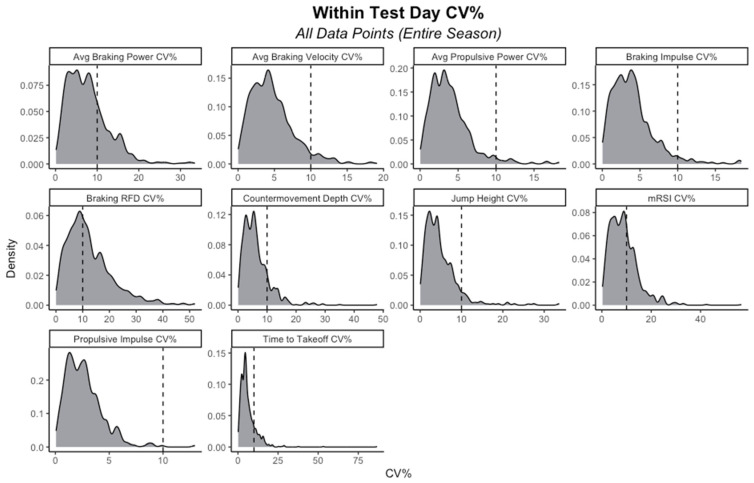
Density plots visualizing the distributions of coefficient of variation (CV%) values for metrics of interest. Note: Vertical dashed lines indicate intra-test-day CV values of 10%; mRSI, modified reactive strength index; RFD, rate of force development.

While different researchers choose different means of quantifying and labeling CMJ phases, in this study, in line with manufacturer guidelines, the CMJ was divided into an unweighting phase, braking phase, propulsive phase, flight phase, and landing phase [15]. The braking phase was defined as the phase starting once the athlete’s system mass returned to baseline force, following a reduction in force during the unweighting phase, until the end of the braking phase (i.e., minimal center of mass displacement). The propulsive phase started immediately following the braking phase and lasted until takeoff. On individual test-days, the mean of three jump trials was calculated for the metrics of interest. For further data analyses, the average of the three best test-days across the in-season period was used for each force–time metric of interest. Table 1 presents detailed descriptions of the force–time metrics examined in the present study. The metrics were chosen based on the previous literature [3,15,16], combined with the respective strength and conditioning coaches’ interest in monitoring these across the in-season period, and they consist of traditional metrics reflecting overall jump performance (e.g., jump height), as well as kinetic metrics (e.g., braking impulse) and metrics reflecting jump strategy (e.g., time-to-takeoff). More specifically, metrics from the aforementioned subgroups were selected to reflect the braking and propulsive phases, highlighting both the jump strategy and output, making for a comprehensive vertical countermovement jump analysis. Therefore, metrics were chosen and divided into subgroups of traditional metrics, kinetic metrics, and strategy metrics, following suggestions from previous literature [16] Intra-test-day coefficient of variation percentages (CV%) were calculated across all athletes and all test-days over the entire study duration and ranged from 2.6% to 12.9%. Figure 1 presents density plots for all metrics of interest, visualizing the distribution of CV% values.

### 2.4. Measures of On-Court Performance

The researchers collected publicly available relevant measures of on-court success from the teams’ respective statistics websites. Given the fact that individual athlete success within basketball can be multifactorial and is difficult to quantify globally, the minutes played per game over the course of the season (post-season excluded) were used as the measure of on-court performance or contribution. Within each team, the sample was divided into high- and low-minutes groups, using a median-split analysis, to analyze specific group differences based on the selected CMJ force–time characteristics. Again, the strength and conditioning coaches of each team confirmed that the group split generally reflected a distinction between those athletes in the primary playing rotation of the team and those athletes who are either non-scholarship players or players who are rarely part of the regular playing rotation.

### 2.5. Statistical Analyses

All data were examined for normality using the Shapiro–Wilk test. To identify potential differences in CMJ force–time metrics between groups, independent-sample *t*-tests with high vs. low minutes as the grouping variable and CMJ force–time metrics as the dependent variable were utilized. Independent-sample *t*-tests were applied to the whole sample (male and female), as well as for each team (i.e., each sex). To show the magnitude of differences between groups, Hedge’s g effect sizes were calculated. Effect sizes were classified as either negligible (<0.20), small (0.21–0.50), moderate (0.51–0.80), or large (>0.80) [17]. Confidence intervals (95% CIs) were generated for mean differences between groups, as well as for effect sizes, using the ‘esci’ package within the R statistical computing environment and language (v. 4.0; R Core Team, 2020) via the Jamovi graphical user interface. If a force–time metric reached a large between-group effect size (>0.80), a follow-up receiver operating characteristic (ROC) curve analysis was implemented to investigate the respective diagnostic utility of the metrics, and to identify actionable thresholds or cut-off points, distinguishing between the groups. Lastly, individual athletes’ within-test-day coefficient of variation percentage (CV%) values were calculated for all force–time metrics of interest across the entire season, by dividing the within-test-day standard deviation by the within-test-day mean and multiplying it by 100 to generate a percentage. Similarly, to previous research [18], between-group mean values and mean differences for each team were visualized using Gardner–Altman plots [19]. The Gardner–Altman plots were generated in RStudio using the ‘dabestr’ package. Statistical inferences were made using an α level of *p* ≤ 0.05.

## 3. Results

**Figure 2:**
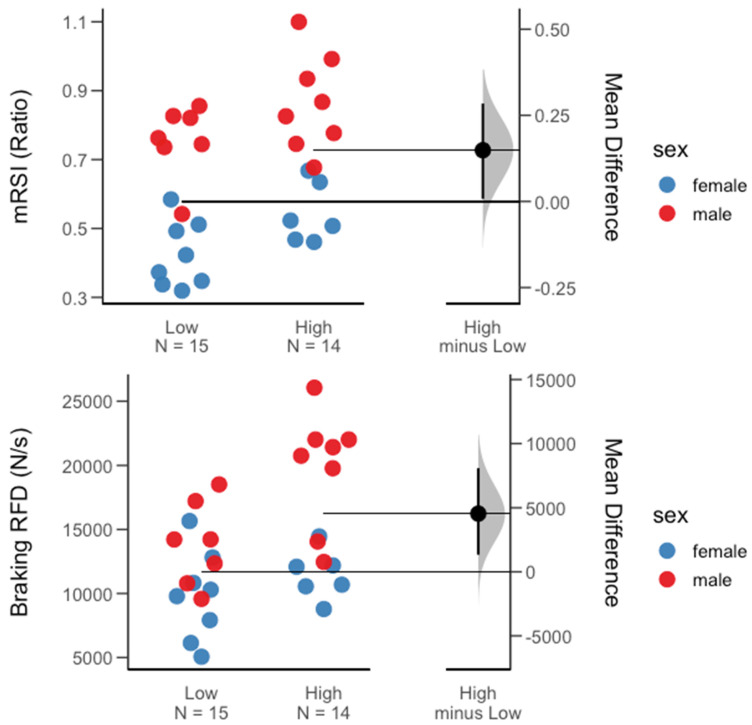
Gardner–Altman plots of the two differentiators with the largest effect size for minutes played within the whole group (male and female). Descriptive plots show the mean and mean difference for and between each group (high vs. low minutes), respectively.

**Figure 3:**
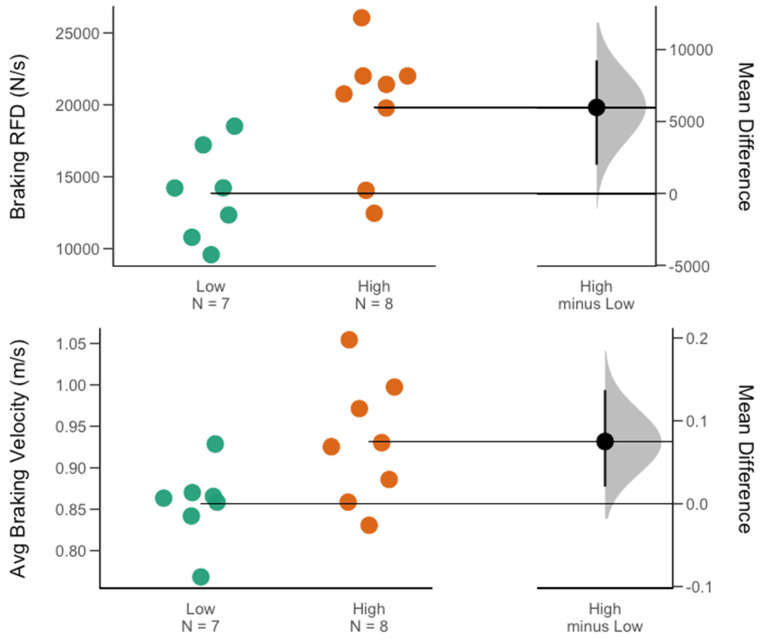
Gardner–Altman plots of the two significant differentiators for minutes played on the men’s team. Descriptive plots show the mean and mean difference for and between each group (high vs. low minutes), respectively.

**Figure 4:**
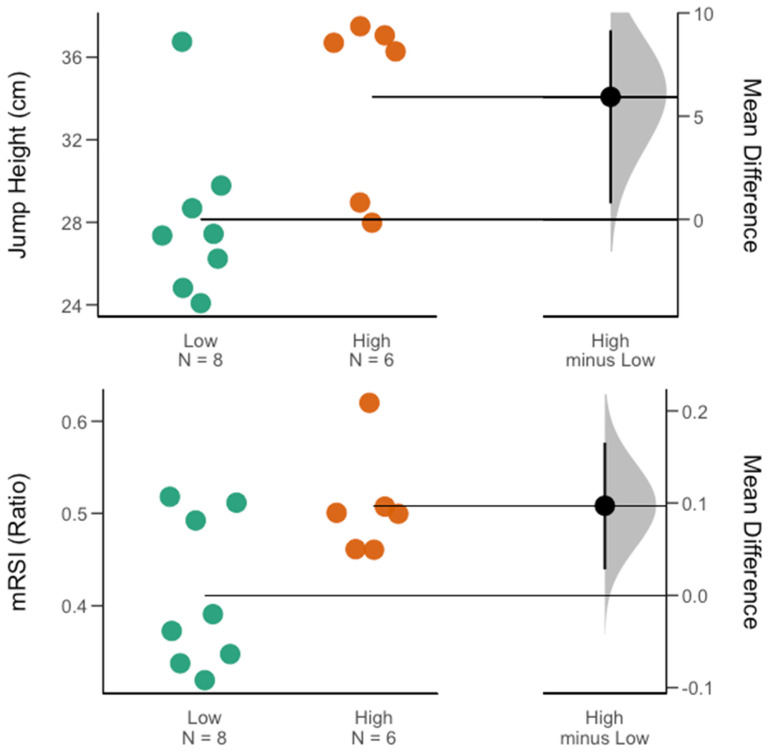
Gardner–Altman plots of the significant differentiators for minutes played on the women’s team. Descriptive plots show the mean and mean difference for and between each group (high vs. low minutes), respectively.

At the overall group level (male and female), with regard to the group of traditional force–time metrics, no significant difference for the high- vs. low-minutes groups was found. However, jump height (ES = 0.66) and mRSI (ES = 0.73) presented moderate to large between-group effect sizes. Within the group of kinetic metrics, a significant difference for high- vs. low-minutes groups was found for the braking RFD (*p* = 0.02, ES = 0.92). Interestingly, with regard to the braking RFD, further gender-specific comparisons revealed that within the men’s team, this significant between-group difference held true (*p* = 0.01, ES = 1.43), while for the women’s team, no significant between-group difference was observed. A significant gender-specific difference was found for the mRSI (*p* = 0.03, ES = 1.24) and jump height (*p* = 0.02, ES = 1.34) in favor of those female athletes receiving more playing time. Further, on the men’s team, besides the braking RFD, athletes in the high-minute group also exhibited significantly greater average braking velocities (*p* = 0.04, ES = 1.12). Table 2 and Table 3 contain descriptive results for between-group comparisons at the overall group level (male and female), as well as for each team. Figure 2, Figure 3 and Figure 4 display between-group differences for significant metrics, as visualized via Gardner–Altman plots. Lastly, Table 4 presents the reader with results from the ROC analysis conducted for metrics that revealed large between-group effect sizes.

## 4. Discussion

The aim of the present study was to investigate the utility of different CMJ force–time characteristics in separating basketball athletes receiving higher and lower amounts of playing time at the NCAA Division-I level of play. These comparisons were carried out across the entire sample involving 14 female and 15 male basketball players, as well as across each team individually, to account for potential gender-specific differences contributing to on-court success. Given the rising popularity in utilizing force platforms and the associated force–time signals to enhance athlete health and development through an individualized approach to training, it is of critical importance to quantify neuromuscular performance qualities underpinning success within the given sport.

For the overall group (male and female), independent-sample *t*-tests suggested a significant difference for high- vs. low-minutes groups for only the braking RFD (ES = 0.92). With regards to mRSI, while non-significant, a moderate to large effect size in favor of the high-minutes group was observed (ES = 0.73). Further, gender-specific comparisons of mRSI showed significant differences between groups for the women’s team (ES = 1.24). While not reaching the level of statistical significance, comparisons within the men’s team suggested a large between-group effect size (ES = 0.83), highlighting the importance of being able to achieve sufficient jump heights while minimizing time-to-takeoff, which has previously been suggested as an overall marker of an efficient jump strategy [20]. Further, within the women’s team, jump height, being a subcomponent of mRSI, also suggested a significant, large between-group difference, in favor of those athletes in the high-minutes group (ES = 1.34).

Previous studies within the basketball realm have reported the importance of rapid force-generating capabilities, especially within the eccentric or braking phase of movements involving a stretch-shortening cycle [21,22,23]. Given the dimensions of the traditional basketball court, athletes are frequently tasked with rapid accelerations and decelerations over shorter distances, as compared to some field-based sports in which playing dimensions are greater (e.g., soccer). While speculative, given the distance and time constraints associated with such rapid decelerations and changes in direction, athletes must be able to generate braking forces in a time-efficient manner. With the braking RFD being the slope of force over a short time period, it is intuitive that such a neuromuscular quality is of importance to basketball athletes.

Interestingly, when team- or gender-specific comparisons were performed, this quality was of greater importance within the group of male athletes. Within the men’s team, braking RFD yielded a large between-group effect size (ES = 1.43) in favor of those athletes in the high-minutes group, while in the women’s team, only a moderate effect size (ES = 0.52) was yielded. The receiver operating characteristic curve follow-up analysis suggested that if male basketball athletes presented with braking RFD magnitudes over 19,778.8 N·s^−1^, in 75% of cases, they were correctly classified as being in the high-minutes group. Actionable thresholds like this may be used by sport science practitioners in benchmarking athletes or tracking their progress over time. Similarly, when examining the average braking velocity (likely a driver of the braking RFD) within the men’s team, between-group comparisons revealed a significant difference, supported by a large effect size (ES = 1.12) in favor of the high-minutes group. On the other hand, within the women’s basketball players, a small between-group effect size was shown, which was actually in favor of those athletes playing fewer minutes. For instance, despite not reaching the level of statistical significance, a small to moderate effect size (ES = −0.42) in favor of those athletes on the women’s team playing fewer minutes was found for average braking power, suggesting further gender-specific relationships between CMJ force–time characteristics and playing time. Based on the findings of the present study, the ability to generate braking force at higher velocities and over shorter time periods carries more relevance in male athletes with regards to on-court performance contributions, as compared to the women’s team. Interestingly, besides average braking power, looking at the braking net impulse, a moderate to large between-group effect size (ES = −0.72) was shown on the women’s team, which was in favor of those athletes within the low-minutes group. It is likely that this finding is attributed to the fact that, while the difference was not statistically significant, athletes within the low-minutes group presented with greater body mass, which was found to be true on both the men’s and women’s teams. While not highlighted in the results, a supplementary ROC analysis suggested that if female athletes presented with a body mass of 80.5 kg or greater, in 88% of cases, they were correctly classified as being in the low-minutes group. This highlights the importance of accounting for differences in body mass when monitoring force–time characteristics in athlete populations displaying wide ranges of anthropometric features.

While speculative, it may be reasonable to suggest that male athletes presented with a more efficient amortization phase, turning large braking impulses into a faster and more forceful propulsive phase, leading to greater takeoff velocities and, therefore, jump heights, as well as mRSI outputs. The amortization phase is defined as being the brief phase when transitioning into and out of the lowest center-of-mass position [24]. This phase and the qualities highlighted within the previous sentences may present practitioners working with female basketball athletes with actionable opportunities to enhance stretch-shortening cycle function. However, readers should also interpret the results from the present study in conjunction with findings from the previous literature highlighting biological differences between genders [25,26]. For instance, previous studies have reported lower RFD values for females, likely due to structural differences in the muscles’ elastic properties [25,26]. These structural differences may help explain why the braking RFD was shown to be of greater importance with regard to the on-court contribution within the men’s team. Further research is warranted focused on investigating whether or not improving qualities such as the braking RFD and braking power within female basketball players leads to greater on-court performance or increases in playing time. Regardless, these findings highlight the importance of acknowledging the gender-specific nature of physical performance, as being related to success within the game of basketball.

While not the primary aim of this study, our results also provide practitioners and other sport scientists with reliability statistics and descriptive data of highly trained male and female basketball players, which may be used for benchmarking or goal-setting purposes. This is of critical importance especially when taking into account that the scientific literature is lacking in data pertaining to elite female athletes.

The strengths of this study may be seen within the highly trained nature of the sample investigated, in conjunction with highlighting gender-specific differences in neuromuscular performance as it relates to on-court performance. Further, the lack of research reports tying physical performance qualities in with measures of sport-specific success presents an opportunity to fill this gap within the literature. However, it must be acknowledged that our data came from a very applied, non-lab-based setting. Coaches and researchers ensured that test procedures were held constant across time, and tests occurred at the same time-point within each training session. However, as a consequence of collecting real-world, in-season data from teams consisting of high-level athletes, it was difficult to control for factors such as overall training volume, practice and game-induced fatigue, and recovery periods between games and CMJ test sessions. This may negatively impact the study’s robustness. While in-season data collection adds ecological validity, it also introduces variability due to factors such as game- and practice-related fatigue. In an attempt to account for this, the authors only analyzed the average of each athlete’s three best test-days across the season. Further, the sample size may be interpreted as a limitation with regards to the external validity and generalizability of the presented findings. Future investigations may aim to replicate methodologies within larger samples, exploring a broader range of neuromuscular performance qualities, in addition to other levels of play. Similarly, while significantly correlated with most basketball-specific player statistics, future studies may also aim to investigate relationships with other, more comprehensive measures of on-court performance. Given the limited sample size, in addition to the number of comparisons, rather than just focusing on *p*-values, readers may also pay attention to between-group effect sizes, as well as confidence intervals for both effect sizes and mean differences. The results should be interpreted in accordance with the limitations that come with studying smaller samples.

Nonetheless, the authors believe that more research must be performed on high-level athletes within real, applied sports settings. Therefore, we believe that our study has the potential to effectively add to the body of literature. It may be worthwhile noting that the median for minutes played per game on the men’s team was 8 min, while it was 22 min on the women’s team. This suggests that there were gender-specific differences with regard to the spread of the measure of on-court contribution used within this investigation. Future investigations may aim to implement similar analyses at different levels of play (e.g., the junior level or professional level).

## 5. Conclusions

In summary, this study highlighted CMJ-extracted measures of neuromuscular performance, capable of distinguishing between levels of playing time within male and female NCAA Division-I basketball players. At the overall group level (male and female), the mRSI and braking RFD showed the greatest between-group magnitudes of difference, with those athletes playing more minutes per game showing greater mRSI values and greater rates of force development during the braking phase of the CMJ. At the team- or gender-specific level, the braking RFD, average braking velocity, and mRSI were shown to be the greatest differentiators between high- and low-minutes groups for the men’s team. Within the women’s team, the jump height and mRSI presented with the greatest magnitude of difference in favor of the high-minutes group, while braking impulse, likely influenced by body mass, presented with the greatest magnitude of difference in favor of the low-minutes group. Practitioners might find the results from this investigation insightful with regards to the selection of neuromuscular performance tests and metrics to monitor basketball athletes. Further, the neuromuscular performance qualities highlighted within this study may aid strength and conditioning coaches in the individualization of their training programs based on force–time data collected. Additional research is warranted to solidify the propositions and suggestions made within this investigation.

## Figures and Tables

**Table 1:** List and definitions of force–time metrics examined in the present study.

**Traditional Metrics (Unit)**	**Definition**
Jump height (cm)	Maximal jump height via impulse—momentum calculation
mRSI (ratio)	Jump height divided by time-to-takeoff
**Kinetic Metrics (Unit)**	**Definition**
Braking net impulse (N·s)	Area under the braking phase of the net force–time curve
Average braking power (W)	Average power generated during the braking phase
Braking rate of force development (N·s^−1^)	The average change in force over time during the braking phase
Propulsive net impulse (N·s)	Area under the propulsive phase of the net force–time curve
Average propulsive power (W)	Average power generated during the propulsive phase
**Strategy Metrics (Unit)**	**Definition**
Average braking velocity (m/s)	Average velocity obtained during the braking phase
Time-to-takeoff (s)	Duration from start of the countermovement until takeoff
Countermovement depth (cm)	Lowest center of mass displacement, transition from braking to propulsive phase

Note: mRSI = modified reactive strength index.

**Table 2:** Independent sample *t*-test comparisons between the high- and low-minutes groups across the whole sample (men and women).

Metric	High Minutes	Low Minutes	Diff (CI)	*p*-Value	ES (CI)
	M ± SD	M ± SD			
Jump height (cm)	45.5 ± 11.1	37.9 ± 11.7	7.89 (−0.83; 16.6)	0.08	0.66 (−0.12; 1.42)
mRSI (ratio)	0.71 ± 0.21	0.56 ± 0.21	0.15 (−0.003; 0.31)	0.06	0.73 (−0.03; 1.47)
Braking net impulse (N·s)	139.5 ± 35.8	140.1 ± 26.3	−0.68 (−24.5; 23.1)	0.95	−0.02 (−0.75; 0.71)
Avg braking power (W)	1462 ± 367	1397 ± 251	65.3 (−303; 173)	0.58	0.20 (−0.53; 093)
Braking RFD (N·s^−1^)	16,098 ± 5715	11,552 ± 3794	4546 (874; 8218)	**0.02**	0.92 (0.14; 1.68)
Propulsive net impulse (N·s)	257 ± 63.7	253 ± 61.2	4.38 (−43.2; 52.0)	0.85	0.07 (−0.66; 0.80)
Avg propulsive power (W)	3287 ± 1002	2986 ± 933	359 (−435; 1039)	0.41	0.30 (−0.43; 1.03)
Avg braking velocity (m·s^−1^)	0.91 ± 0.09	0.87 ± 0.06	0.04 (−0.10; 0.02)	0.29	0.54 (−1.28; 0.21)
Time-to-takeoff (s)	0.66 ± 0.11	0.68 ± 0.09	−0.04 (−0.09; 0.06)	0.68	−0.15 (−0.87; 0.58)
Countermovement depth (cm)	33.0 ± 6.9	33.7 ± 1.61	−0.71 (−0.64; 0.82)	0.80	−0.09 (−0.64; 0.82)

Note: A bold *p*-value indicates a significant between-group difference. Avg, average; RFD, rate of force development; mRSI, modified reactive strength index; ES, effect size (Hedge’s g); Diff, mean difference; CI, 95% confidence interval.

**Table 3:** Comparisons between high- and low-minutes groups for their respective teams (male vs. female).

Metric	High Minutes	Low Minutes	Diff (CI)	*p*-Value	ES (CI)
	M ± SD	M ± SD			
Male					
Jump height (cm)	53.4 ± 6.61	48.8 ± 6.57	−4.54 (−2.83; 11.9)	0.21	0.65 (−0.38; 1.92)
mRSI (ratio)	0.87 ± 0.14	0.76 ± 0.10	0.11 (−0.03; 0.25)	0.11	0.83 (−0.19; 2.16)
Braking net impulse (N·s)	162 ± 29.4	154 ± 16.3	7.66 (−19.5; 34.8)	0.55	0.30 (−0.77; 1.48)
Avg braking power (W)	1623 ± 392	1427 ± 271	195 (−152; 542)	0.25	0.59 (−0.44; 1.85)
Braking RFD (N·s^−1^)	19,819 ± 4460	13,841 ± 3246	5978 (1565; 10,390)	**0.01**	1.43 (0.41; 2.97)
Propulsive net impulse (N·s)	303 ± 40.2	306 ± 29.8	−2.40 (−42.4; 37.6)	0.90	−0.06 (−1.19; 1.04)
Avg propulsive power (W)	4074 ± 416	3842 ± 479	232 (−267; 731)	0.33	0.49 (−0.56; 1.71)
Avg braking velocity (m·s^−1^)	0.93 ± 0.07	0.86 ± 0.05	0.08 (0.004; 0.15)	**0.04**	1.12 (0.10; 2.54)
Time-to-takeoff (s)	0.71 ± 0.09	0.73 ± 0.07	−0.01 (−0.11; 0.08)	0.78	−0.14 (−1.28; 0.96)
Countermovement depth (cm)	31.3 ± 4.62	29.2 ± 2.38	2.12 (−2.08; 6.32)	0.30	0.53 (−0.51; 1.77)
Female					
Jump height (cm)	34.1 ± 4.38	28.1 ± 4.0	5.64 (−10.8; −1.07)	**0.02**	1.34 (−0.12; 2.39)
mRSI (ratio)	0.51 ± 0.06	0.41 ± 0.08	0.10 (0.01; 0.18)	**0.03**	1.24 (0.05; 2.38)
Braking net impulse (N·s)	110 ± 16.0	128 ± 28.1	−18.2 (−46.2; 9.78)	0.18	−0.72 (−1.80; 0.39)
Avg braking power (W)	1249 ± 184	1371 ± 320	−122 (−48.9; 469)	0.42	−0.42 (−0.66; 1.48)
Braking RFD (N·s^−1^)	11,139 ± 2326	9550 ± 3152	1588 (−1750; 4927)	0.32	0.52 (−0.57; 1.59)
Propulsive net impulse (N·s)	196 ± 19.9	207 ± 38.7	−11.1 (−48.7; 27.1)	0.55	−0.31 (−1.37; 0.76)
Avg propulsive power (W)	2240 ± 250	2237 ± 413	2.73 (−420; 414)	0.99	0.01 (−1.05; 1.07)
Avg braking velocity (m·s^−1^)	0.76 ± 0.07	0.79 ± 0.10	−0.02 (−0.13; 0.08)	0.65	−0.24 (−0.90; 1.47)
Time-to-takeoff (s)	0.72 ± 0.08	0.77 ± 0.09	−0.04 (−0.06; 0.15)	0.37	−0.47 (−1.76; 0.63)
Countermovement depth (cm)	26.9 ± 4.35	28.6 ± 5.32	−1.68 (−4.13; 7.48)	0.54	−0.32 (−1.57; 0.80)

Note: A bold *p*-value indicates a significant between-group difference. Avg, average; RFD, rate of force development; mRSI, modified reactive strength index; ES, effect size (Hedge’s g); Diff, mean difference; CI, 95% confidence interval.

**Table 4:** Gender-specific receiver operating characteristic curve analysis data.

Metric	Gender	Specificity	Sensitivity	AUC	Cut-Off (Threshold)
Braking RFD (N·s^−1^)	Male	0.71	0.75	0.86	19,778.8
mRSI (ratio)	Male	0.57	0.63	0.73	0.87
Avg braking velocity (m·s^−1^)	Male	0.86	0.75	0.79	0.87
Jump height (cm)	Female	0.88	0.67	0.85	29.0
mRSI (ratio)	Female	0.63	0.67	0.75	0.46

## Data Availability

The data presented in this study are available on request from the corresponding author.
